# Moral Blindness – The Gift of the God Machine

**DOI:** 10.1007/s12152-016-9272-9

**Published:** 2016-07-20

**Authors:** John Harris

**Affiliations:** University of Manchester, Manchester, UK

**Keywords:** Moral enhancement, The Value of Life, Feminization, The all female world

## Abstract

The continuing debate between Persson and Savulescu and myself over moral enhancement concerns two dimensions of a very large question. The large question is: what exactly makes something a moral enhancement? This large question needs a book length study and this I provide in my How to be Good, Oxford 2016. (JH 2016). In their latest paper Moral Bioenhancement, Freedom and Reason take my book as their point of departure and the first dimension of the big question they address is one that emphasizes a distinction, not highlighted in their original 2008 paper, between a moral enhancement that will ensure an improvement in morality and one that will simply make people more motivated to be moral. The second issue concerns whether anything that would be a “moral enhancement” properly so called, could involve denying moral agents the very possibility of autonomously choosing to try to be good. In this response, although P&S cover a number of other related issues, I shall concentrate on these two points.

**What is Moral Enhancement?**

The continuing debate between Persson and Savulescu (P&S) and myself over moral enhancement concerns two dimensions of a very large question. The large question is: what exactly makes something a moral enhancement? This large question needs a book length study and this I provide in my *How to be Good,* Oxford 2016. [1]. In their latest paper *Moral Bioenhancement, Freedom and Reason* P&S take my book as their point of departure and the first dimension of the big question they address is one that emphasizes a distinction, not highlighted in their original 2008 paper, between a moral enhancement that will **ensure** an improvement in morality and one that will simply make people more **motivated** to be moral. The second issue concerns whether anything that would be a “moral enhancement” properly so called, could involve denying moral agents the very possibility of autonomous moral choice.

In this response,^1^ although P&S cover a number of other related issues, I shall concentrate on these two points.

P&S initially predicated their whole, veritable Norse Saga, of moral enhancement on the absolute necessity to save the world from even a lone maniac or idiot who might by accident or design wreak literally limitless harm. Because of the scale of the harm that might be done by such an outlier they originally argued that countermeasures in the form of moral enhancement would have to be foolproof, universal and obligatory. They have since back-peddled considerably (although they seem unaware of this) and now talk of moral enhancement as matter, not of **ensuring** and **controlling** outcomes, but of improving moral **“motivation”,** which word, or its variants, appears six times in their latest paper.

They now say two mutually contradictory things. The first is that we must motivate people to choose the moral path, not that we must absolutely prevent them from choosing the immoral one. But they also continue to defend the desirability of a “God Machine” or its equivalent which while (they now claim) does not itself amount to a moral enhancement properly so-called, none-the-less constitutes an enhancement that, if it could be invented, would make the world a better place precisely because of its ability to control what it would deem to be seriously immoral behaviour. This still seems to me both false in fact, because it would not make the world a better place, and a literally monstrous perversion of any minimal sense of what might constitute a moral enhancement.

They make a point of saying that I now seem to agree with them but, I am afraid, it is they that, to large extent, have come to agree with me, at least on one of these two points.

Lets see what’s going on.

They say (2008: 174):

*Even if only a tiny fraction of humanity is immoral enough to want to cause large scale harm by weapons of mass destruction in their possession, there are bound to be some such people in a huge human population… unless humanity is extensively morally enhanced.*

*A moral enhancement of the magnitude required to ensure that this will not happen is not sufficiently possible at present and is not likely to be possible in the near future…*

*until effective means of moral enhancement are found and applied…*

*Genetic engineering of smallpox could create a new strain which would wipe out all or most of humanity.*

And P&S ([2]: 174) conclude that:

*If safe moral enhancements are ever developed, there are strong reasons to believe that their use should be obligatory…That is, safe, effective moral enhancement would be compulsory.*

Hence the attraction of the God Machine. It is important to be clear that P&S apparently believe, or once believed (maybe no longer?) that it was simply not enough to develop forms of moral enhancement that involved the creation or modification of people so that they were more “motivated” to do good. Public and personal safety demands, according to the previous P&S, that as far as is humanely possible the sort of moral enhancement that could achieve this had to be, in their own words (2008, 174), “*of a magnitude to* ***ensure*** *that this will not happen”* and accordingly that the implementation of moral enhancement that could achieve this “*should be* ***obligatory*** *… That is, safe, effective moral enhancement would be* ***compulsory*** *”.*

In clarification of how this could, in theory, be both universal and compulsory they offered, the implausible, but on their view optimal, solution if only it could be realized: a “God Machine” [3] which would have a purpose built “freaky mechanism” to prevent wrongdoing. They now admit that the God machine would not be a moral enhancement though it would, they believe, be a very good thing.

They say: (2016: 2).

*“Imagine, however, that there is a freaky mechanism in your brain which would have kicked in if you had been in the process of making, … a decision to do something which is morally wrong. The mechanism would then irresistibly have made you decide to do the morally right thing. Hence, you are not free to fall, i.e. you cannot avoid deciding to do the morally right thing.” This freaky mechanism lead to the building of a God Machine to prevent serious wrongdoing.”*

I had objected in my book [1] and elsewhere, that morality was basically a matter of choosing what is for the best all things considered, not simply being well motivated or pro-social; in short that to be good is not simply happening to do no evil but choosing for a reason, choosing on the basis of evidence and argument, not to do wrong.

S&P (2016: 2–3) now admit:

*“The freaky mechanism does not count as moral enhancement in our vocabulary, since it does not enhance your motivation to do what is morally right. Rather, it deprives you of your freedom – and even ability – to decide to do and do what is wrong.”*

But notice that this is just what S&P originally claimed that moral enhancement should aim at, namely to “*ensure*” (i.e. guarantee) that wicked cataclysmic events would not be chosen and to make assurance doubly sure, that such measures had to be in the words of S&P “*obligatory*” and “*compulsory*”.

S&P now, more that a little disingenuously say: *To sum up, it seems to us that if Harris were to cash out the metaphors quoted above, he would land in our position that moral enhancement in the sense of enhancement of moral motivation is necessary to make us act morally more often. Enhancement of moral motivation is not inimical to reasoned judgements about morally relevant matters, like suffering, but rather presupposes them.* (2016: 2).

S&P go on to remind readers that their God Machine (GM) is just the freaky mechanism universalized and that: ‘*GM only restricts freedom or ability to act to a small extent*” (2016: 4–5) and they conclude by re-asserting their literal “espousal” of GM as a good idea if it could only be created: *“We espouse the way of MB because, though long and difficult, it may be necessary to this end”* the end being *“to anticipate criminal actions, e.g. terror attacks, and nip them in the bud, and GM is a particularly effective way of doing that.”* (2016:6) GM may only “restrict freedom or ability to act to a small extent” but as I have argued where and to the extent that it does it cannot count as a moral enhancement.

## Could we Rely on a God: Machine or Person?

S&P rightly point out that the Gods with whom (which?) we are familiar are far from perfect, and this, while broadly right, is clearly an understatement. But all the Gods (except those created by S&P?) are either fictional or mythical, or if they are not their differences cancel each other out. It is important to be wary of how far their, admittedly freaky, God should be believed in or trusted if it were ever to be created by the would-be God-makers P&S. I have argued [1] that it is not clear that even a God Machine with powers of thought similar to those of any known Gods could make the relevant distinctions required by the P&S formula.

I have also argued [1] that any GM worthy of the name would have to be capable of thinking for itself and could not be relied on simply to follow a programme devised by P&S. Suppose, for example, the God machine talked itself into the idea that humanity is led into moral temptation by that which delights the eye and by the pleasures of the flesh, and so engineers all future children to be blind and incapable of sexual pleasure. The malign, malicious mechanism that the GM is might just be capable of talking itself into such an intervention, much in the same way as some other moral monsters have for centuries made related interventions, often in the name of a God like the GM invented by man, to for example, deprive women of analogous possibilities by genital cutting.

If the senses of sight and sexuality were to be removed at a stage of development prior to the development of sight or sexual desire, future generations would not know what they were missing, and so by the sort of reasoning used by P&S, would neither be rendered un-free by the inability to experience these sensations any more than would those ‘doctored’ by the God Machine into a pro-sociality they had not chosen to exhibit.

To take a more realistic case: suppose your passport has been, unbeknown to you, cancelled so you are not free to travel – you would be turned back at border control or when attempting to board a plane. Even when you are not thinking of using it, you are proud of your passport and the freedom and re-assurance it gives you, but this has been rendered illusory. It would be odd to think that no harm had been done so long as you never travelled or became aware of the freedom limiting decision that had been taken against you.

It is essential to the line run by S&P that all these Frankfurt-style cases show that the principle of alternative possibilities is false, but this is, at the very least, highly controversial [4]. The Stanford Encyclopedia of Philosophy [5] for example, glossing John Locke’s famous discussion of “locked room” cases (the model for Frankfurt style cases) where someone stays voluntarily in a locked room and never discovers that he had in fact no “alternative possibility” to leave, notes:

*“Voluntariness, then, is not necessary for freedom; but it is also not sufficient for freedom, as Locke’s “locked room” …The man in the locked room wills to stay and talk to the other person in the room, and this volition is causally responsible for his staying in the room: on Locke’s theory, his remaining in the room is, therefore, voluntary. But the man in the locked room “is not at liberty not to stay, he has not freedom to be gone” (E1-5 II.xxi.10: 238).”* (http://plato.stanford.edu/entries/locke-freedom/. )

Many seriously ill people hoard pain killers so that they have a lethal dose to hand in case of need. [6, 7] Suppose, unbeknownst to them, the God machine has substituted sugar pills to deprive them of their ability, their freedom, to decide the time and manner of their own death. Just the sort of thing P&S’s moralistic machine would do! And yet P&S seem to believe that no harm would have been done and no threats to freedom would have been made so long, that is, as they die without ever choosing to have recourse to their now useless safety net. I hate to risk yet another accusation of chauvinism (see below) by quoting that very English writer William Shakespeare. Shakespeare has Cassius explain that his ability to kill himself, when and if he so chooses, is a guarantee of ultimate liberty:

*But life, being weary of those worldly bars,*

*Never lacks power to dismiss itself.*

*If I know this, know all the world besides,*

*That part of tyranny that I do bear*

*I can shake off at pleasure.*

William Shakespeare *Julius Caesar Act I. Sc. III.*

S&P seem to think that capacities like autonomy and liberty, or more modest ones like the ability to speak Italian, are capacities only possessed when actually exercised and hence are only lost when we try and fail to exercise them. Suppose the God machine capriciously took against the Italian language and wiped out my ability to speak that beautiful tongue (albeit badly) while I slept. Would I only have been wronged when I tried but failed to speak Italian, and if I had died without ever having the occasion to try, would I have suffered no deprivation, no harm, no insult and no injury whatsoever?

I judge the answer to be clear, and I am afraid, one which renders P&S’s account implausible.

## Is Harris as morally Bankrupt as the God Machine?

I end by noting and responding to two complaints S&P make of my own moral conduct or character, one of which, my penchant for literary quotations, has already been mentioned.

*“Harris is fond of sprinkling his text with quotations from literary authors, but they are all (with the exception of some classical) British: Shakespeare, Milton, Byron, Auden, and Golding. If his choice of literature had been less chauvinistic, it might have included Fyodor Dostoevsky’s The Brothers Karamazov and Ivan Karamazov’s diatribe against God.”* (2016: 4)

Concerning my fondness for acknowledging the wisdom and power of literary and artistic sources as well as more academic or ‘philosophical’ ones, I am certainly guilty as charged. However the charge of chauvinism is both tendentious and entirely unwarranted by the available evidence, evidence of which S&P must have been aware.

I am unclear why classical sources are to be discounted, (perhaps because none of them could be British?) but even, for the sake of argument allowing this, my book contains reference to or quotation from no less than18 non-British, non-classical and non-university literary and artistic sources, as a brief glance at the Index would have revealed. It is gratifying I am sure for both S&P as well as for myself, to be able to record that S&P managed to find just one literary source to quote back at me.

Lastly, S&P also seem to be claiming some sort of originality for the idea that women tend to have a rather better record for goodness, or more accurately, that they demonstrate a much more restricted predilection for evil than do men, and that one strategy for moral improvement might involve the feminization of the world. They seem to think I should have acknowledged their priority for noting this (2016: 2):

*On the basis of this, we suggested that MB could consist in making men in general more like women in general in respect of the capacity for sympathy. Without acknowledging any awareness of our prior discussion, Harris proposes a ‘radical feminization of men’ (2016: 85) as a way of achieving moral enhancement. As we explicitly did.*

I freely admit to a failure here, I was indeed aware of the discussion to which they refer and it is true that I chose not to further discuss or cite their previous, but not prior, discussion. This is in part because I had myself discussed at length analogous possibilities in my book *The Value of Life,* published way back in 1985. I then devoted half of Chapter 8 of that book to a discussion of the feminization of the world. I there imagined that a resurgent feminism believed:

*...a good measure of the evils of the world had been brought about both by the dominance of men, and by the dominance of certain distinctively (though not exclusively) male characteristics. Suppose they believed, as many women do, that men were on the whole more egocentric, aggressive, competitive and intolerant than women, and that these features made them in turn more violent, insensitive and perhaps more callous. It might then seem a rational and progressive step to attempt to create a society from which these disastrous characteristics, and the characters that possessed them, had been eliminated. …* ([8]: 166)

*The rationale of this society might be the simple proposition that reform required not the development, acceptance and implementation of a new political and moral theory, but rather required a new type of citizen. And the society which produced such citizens would be founded on and embody, not a political so much as, say, a eugenic theory.*

*This might not be so bizarre or so crazy a view as might at first appear. One of the fears most commonly expressed about attempts to change the human personality by genetic engineering, so that more desirable features would become dominant is simply that we cannot predict what other undesirable changes would be consequent on such an attempt. This fear, particularly, characterised much of the discussion about the desirability of eliminating aggression from the human psyche. It was pointed out that, while aggression was undesirable, it might not be possible to eliminate such an emotion without also destroying the basis of other more desirable emotions, such as love. What would love be like if it did not involve some aggression towards anyone or anything that would destroy our loved ones, for example? Now in proposing an all-female society, we would not be faced with quite the same problems. For one thing we know what women are like--we would not be contemplating the creation of new or radically altered human beings*…([8]: 167-168)

As will be clear from the passages just quoted, my discussion prefigured both the possibility of genetic or other bioenhancement to effect moral improvement of the sort which has now, decades later, been espoused and allegedly invented by S&P. I then went on to outline one possible and morally defensible scenario or thought experiment for achieving a radical feminization of the world’s future population. I note, but do not complain, that S&P do not acknowledge any awareness of my prior discussion, which anticipated their ‘invention’ of moral bioenhancement by 30 or so years, in a book that is still widely available today. (https://www.amazon.co.uk/s/ref=nb_sb_noss?url=search-alias%3Daps&field-keywords=John+Harris+%22The+Value+of+Life%22. ).

## Footnotes

1 I am grateful to David Lawrence and to Neil Levy for helpful comments throughout.
